# *Candida auris* rates in blood culture on the rise: results of US surveillance

**DOI:** 10.1128/spectrum.02216-23

**Published:** 2023-08-25

**Authors:** Brooklyn A. Noble, Kristen L. Jurcic Smith, Jay D. Jones, Ben W. Galvin, Tristan T. Timbrook

**Affiliations:** 1 Data Science, bioMérieux, Salt Lake City, Utah, USA; 2 US Medical Affairs, bioMérieux, Salt Lake City, Utah, USA; 3 Global Medical Affairs, bioMérieux, Salt Lake City, Utah, USA; 4 Department of Pharmacotherapy, University of Utah College of Pharmacy, Salt Lake City, Utah, USA; University of Chicago, Chicago, Illinois, USA

**Keywords:** *Candida auris*, multidrug resistance, bloodstream infection, surveillance network, public health

## Abstract

*Candida auris* is an emerging pathogen that poses a significant public health risk. Its multidrug resistance has led to high mortality, making rapid detection crucial for effective treatment and prevention of transmission. Recent data from the Centers for Disease Control and Prevention indicate a substantial increase in *C. auris* cases in the United States, with a 95% rise in 2021. To provide an update on the detection rates of *C. auris*, we analyzed blood culture results from a near real-time cloud-based surveillance network, BioFire Trend. From January 2021 to April 2023, 34 *C. auris* detections were observed. The analysis showed a notable increase in detections in 2023 compared to previous years. The detection rate in 2023 was higher in all four US Census Regions, except for the Northeast, where it remained constant. The findings emphasize the continuous rise in *C. auris* cases and highlight the importance of near real-time surveillance systems in monitoring this emerging pathogen.

## LETTER

*Candida auris* is an emerging pathogen and public health risk. Systemic infections of *C. auris* are associated with high mortality rates due to multidrug resistance in approximately 50% of isolates (1). Rapid detection of *C. auris* is important for both clinicians to provide appropriate therapy and infection preventionists to limit transmission (2).

According to the Centers for Disease Control and Prevention (CDC), *C. auris* cases in the United States (US) have risen in recent years (2019–2021), with a dramatic 95% increase in 2021 (3). The objective of this study was to provide an update on temporal and geographic changes in *C. auris* detection rates since 2021 using a cloud-based near real-time surveillance network.

The BIOFIRE Blood Culture Identification 2 (BCID2) Panel (bioMérieux, Salt Lake City, Utah) tests for 43 targets associated with bloodstream infections, including *C. auris*, and was launched in mid-2020 (4). For participating healthcare facilities, deidentified BIOFIRE BCID2 patient results are captured by the BIOFIRE Syndromic Trends (TREND) database, a cloud-based pathogen surveillance network (5). We analyzed this database for positive detections of *C. auris,* which included a US cohort of over 90 facilities from 2021 to 2023. Each result in the database likely reflects a unique culture, not necessarily a unique patient. In an attempt to report only unique patients, we limit our consideration to a single *C. auris* detection per facility and a 3-day window.

**Fig 1:**
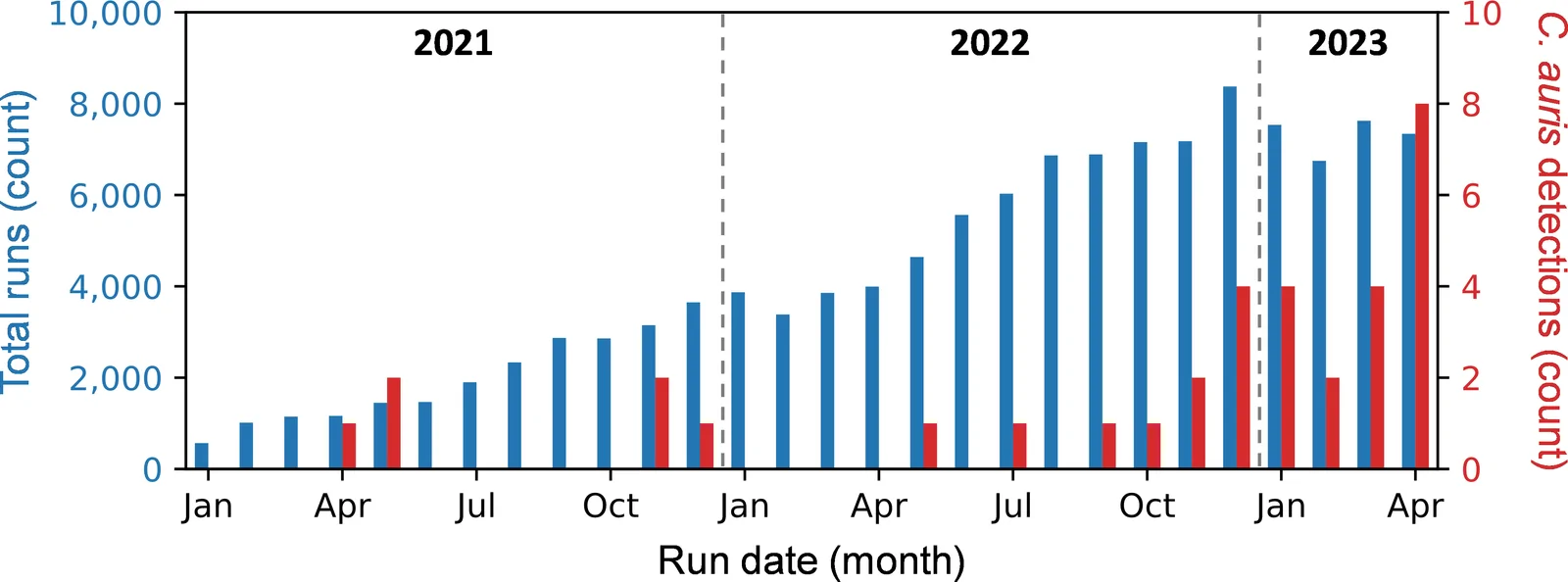
The total number of BIOFIRE BCID2 runs in BIOFIRE TREND (left axis, blue) and the number of those runs with a positive detection for *C. auris* (right axis, red), aggregated monthly.

From January 2021 to April 2023, we observed 34 *C*. *auris* detections. Fig. 1 shows the total number of runs in the database (left axis, blue) and the number of those runs with a positive detection for *C. auris* (right axis, red), aggregated monthly. From Fig. 1, we observe a marked increase in *C. auris* detections in 2023. The average *C. auris* detection rate was 0.025% with a 95% binomial confidence interval of [0.0093%, 0.055%] in 2021, 0.015% [0.0071%, 0.027%] in 2022, and 0.062% [0.036%, 0.097%] in 2023 (January–April). This lends evidence to a statistically significant increase in the *C. auris* detection rate from 2022 to 2023 (January–April).

**Fig 2:**
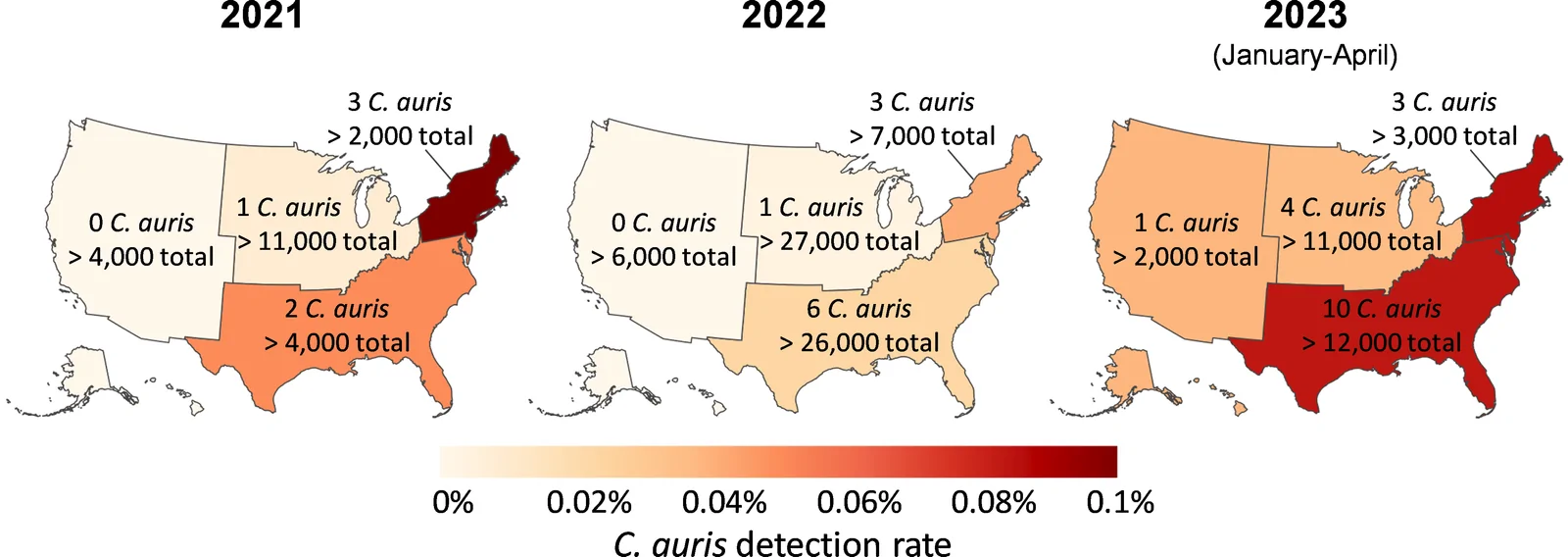
US Census Region choropleth maps of *C. auris* detection rates for 2021, 2022, and 2023 (January–April). For each Census Region and year, labels indicate the total number of BIOFIRE BCID2 runs in BIOFIRE TREND and the number of those runs with a positive detection for *C. auris*.

*C. auris* detections were observed from 15 facilities, spanning 12 states and all 4 US Census Regions. Figure 2 shows yearly US Census Region choropleth maps of *C. auris* detection rates with labels that indicate the total number of runs in the database and the number of those runs with a positive detection for *C. auris*. From Fig. 2, we observe that the *C. auris* detection rate and number of *C. auris* detections were higher in 2023 than in 2021 or 2022 for all four Census Regions except for the Northeast, where the number of *C. auris* detections was constant from 2021 to 2023. The same results were obtained when we limited our consideration to a single *C. auris* detection per facility and a 6-day window. Similar results were obtained when the *C. auris* detections considered were not restricted.

In summary, *C. auris* detections have substantially increased in the last year. Our data show an increase in *C. auris* detections among users of the BIOFIRE BCID2 Panel in 2023, which suggests the rate of *C. auris* detections has continued to rise in the US since the CDC reported an increase through 2021. Near real-time surveillance, as provided by BIOFIRE TREND, can be easily tracked as an adjunctive source of surveillance for this emerging pathogen.

## Data Availability

The data obtained by bioMérieux are subject to the terms and conditions of a Data Use Agreement (DUA) by and between bioMérieux and each facility participating in the TREND program. If a data set is requested, bioMérieux will review such request internally to ensure that any disclosure does not conflict with bioMérieux’s obligations and restrictions set forth in the DUA. Code available upon reasonable request.
